# Incidence, Clinical Significance, and Longitudinal Signal Characteristics of Ischemic Lesions Related to Diagnostic Cerebral Catheter Angiography

**DOI:** 10.1007/s00270-023-03415-z

**Published:** 2023-03-29

**Authors:** David Schinz, Thomas Zimmermann, Jens Göttler, Dominik Sepp, Claus Zimmer, Tobias Boeckh-Behrens, Jan S. Kirschke, Kornelia Kreiser, Hans Liebl

**Affiliations:** 1 Department of Diagnostic and Interventional Neuroradiology, School of Medicine, Klinikum rechts der isar, Technical University of Munich, Ismaninger Street 22, 81675 Munich, Germany; 2 Department of Radiology/Neuroradiology, RKU, Universitäts- und Rehabilitationskliniken Ulm, gGmbH, Oberer Eselsberg 45, 89081 Ulm, Germany; 3 Department of Radiology/Neuroradiology, BGU, Berufsgenossenschaftliche Unfallklinik, Murnau, Professor-Kuentscher-Straße 8, 82418 Murnau Am Staffelsee, Germany

**Keywords:** Silent ischemia, Ischemia, Stroke, Neuroangiography

## Abstract

**Purpose:**

Cerebral DSA is a routine procedure with few complications. However, it is associated with presumably clinically inapparent lesions detectable on diffusion-weighted MRI imaging (DWI lesions). However, there are insufficient data regarding incidence, etiology, clinical relevance, and longitudinal development of these lesions. This study prospectively evaluated subjects undergoing elective diagnostic cerebral DSA for the occurrence of DWI lesions, potentially associated clinical symptoms and risk factors, and longitudinally monitored the lesions using state-of-the-art MRI.

**Materials and Methods:**

Eighty-two subjects were examined by high-resolution MRI within 24 h after elective diagnostic DSA and lesion occurrence was qualitatively and quantitatively evaluated. Subjects’ neurological status was assessed before and after DSA by clinical neurological examination and a perceived deficit questionnaire. Patient-related risk factors and procedural DSA data were documented. Subjects with lesions received a follow-up MRI and were questioned for neurological deficits after a median of 5.1 months.

**Results:**

After DSA, 23(28%) subjects had a total of 54 DWI lesions. Significantly associated risk factors were number of vessels probed, intervention time, age, arterial hypertension, visible calcified plaques, and less examiner experience. Twenty percent of baseline lesions converted to persistent FLAIR lesions at follow-up. After DSA, none of the subjects had a clinically apparent neurological deficit. Self-perceived deficits were nonsignificantly higher at follow-up.

**Conclusion:**

Cerebral DSA is associated with a considerable number of postinterventional lesions, some persisting as scars in brain tissue. Presumably because of the small lesion size and inconsistent location, no clinically apparent neurological deficits have been observed. However, subtle self-perceived changes may occur. Therefore, special attention is needed to minimize avoidable risk factors.

**Supplementary Information:**

The online version contains supplementary material available at 10.1007/s00270-023-03415-z.

## Introduction

Endovascular cerebral digital subtraction catheter angiography (DSA) is a widely used procedure to evaluate a variety of intracranial vascular pathologies like aneurysms, vascular malformations, or tumors. It remains the diagnostic gold standard to diagnose vascular disease and provides the option for direct subsequent physical intervention. As an invasive catheter procedure, it is associated with complications such as groin hematoma, contrast-induced nephropathy, and, in rare cases, embolic events that can lead to neurological deficits or even death [1]. Neurological complications have been reported to be around 0.5–3.2%, whereby only a small fraction of these resulted in permanent neurological impairments [1–4].

However, studies have shown the presence of embolic ischemia-like lesions on diffusion-weighted MRI (DWI) after catheter procedures. These lesions were found more frequently than expected compared with the low amount of clinically reported postprocedural complications [5–11]. Studies using Doppler sonography during the catheterization have shown embolic-like signals in up to 29% of DSA culminating at the time of injection [5–11]. In clinical routine, these lesions are rarely reported or stay undiagnosed. Consequently, their impact on the patient’s health remains unclear and the lesions are referred to as ‘clinically silent’ [12, 13]. Subtle neurological impairments may not immediately be reported after DSA or could occur with delay [3, 14]. No causality for cerebral microemboli and cognitive impairment has been found, nor has it been ruled out [15].

To further characterize the risk profile of DSA, we performed a prospective analysis investigating the incidence of DWI lesions using high-resolution MRI in subjects undergoing elective cerebral diagnostic DSA at our institution. Subjects presenting with a DWI lesion at baseline were scheduled for a follow-up MRI scan to investigate longitudinal signal development. Neurological status was examined and subjective well-being was evaluated by a questionnaire immediately before DSA, after DSA, and at follow-up.

We hypothesize that (1) lesion incidence is higher with the use of high-resolution isotropic DWI sequences than in previous studies; (2) lesion occurrence is associated with DSA or examiner-related risk factors; and (3) lesions persist and may be associated with measurable clinical impairment or self-perceived neurological deficits.

## Materials and Methods

### Study Cohort

Subjects scheduled for diagnostic cerebral DSA between June 2015–2020 were asked to voluntarily participate in this study receiving additional MR imaging free of charge at our institution. Participants received an MRI scan within 24 h after DSA. Study participants presenting with lesions in the baseline MRI exam were offered another voluntary MRI scan free of charge a minimum of 2 months after the baseline scan.

Clinical DSA indications included examination of suspected or known aneurysm or vascular malformation. Subjects with a history of recent head trauma, acute stroke, bleeding, or tumor were excluded to account for potentially confounding lesions. Further exclusion criteria were MRI contraindications according to our institutional and national regulations. Subjects were free to end participation at any time during the study.

This study was approved by the local ethics committee following the guidelines of the declaration of Helsinki. A total of 82 subjects undergoing DSA could be included and signed informed consent.

### DSA and MR Imaging Data

DSA procedures were clinically indicated independent of this study and carried out according to our institutional standards using manual iodine contrast injection (Imeron® 300; Bracco Imaging Deutschland GmbH, Konstanz) and heparinized saline drip (1400 I.E. per liter). Contrast syringes were manually filled from a small bowl of contrast medium and no air filters were used. As part of the study analysis, the number of cerebral vessels examined, fluoroscopy duration, amount of contrast agent, and type of catheters used were documented. DSA was performed by a total of eight senior neuroangiographers, five experienced neuroangiographers, and five novice neuroangiographers.

After DSA, all participating subjects received MRI within 24 h. MRI data were obtained using a Philips Achieva 3 Tesla and Philips Ingenia 3 Tesla system.

For DWI, a B value of 1000 s/mm^^2^, a TR of 7,637 ms, and a TE of 55 ms were used with a resulting isotropic resolution of 2 mm allowing for the detection of small DWI lesions. In addition, coronary DWI was performed to validate potential lesions with a B value of 1000 s/mm^^2^, a TR of 2432 ms, and a TE of 75 ms at a slice thickness of 3 mm. For 3D-FLAIR images, a TR of 4800 ms, TE of 300 ms, and an echo train length of 170 were used, acquired in isotropic 1 mm voxels. Follow-up MRI scans included additional susceptibility-weighted imaging (SWI) to account for potential microbleeding with a TR of 51 ms, TE of 30 ms, and a slice thickness of 1.5 mm.

Image readings were carried out in consensus by two neuroradiologists with 8 years and 3 years of clinical experience, blinded for DSA procedure data, clinical history, and comorbidities. If a lesion presented, the subject was scheduled for a follow-up head MRI within 2–24 months after DSA. Lesion size was measured with the ABC/2 Method by multiplication of maximal lesion diameter and maximal orthogonal diameter times the number of slices with visible lesion hyperintensity and slice thickness.

### Neurological Assessment

Before and after cerebral DSA, a medical interview and neurological examination were performed by the admitting physician. To examine subtle self-perceived changes by the enrolled subjects, we conducted an additional investigator-guided normative questionnaire, the perceived deficits questionnaire (PDQ) [16]. The questionnaire was performed by a specifically trained medical student before the DSA and after DSA before dismissal from the institution, as well as at the follow-up appointment. The PDQ offers the examination of four major traits: attention/concentration, retrospective memory, prospective memory, and planning/organization. It was primarily developed for people suffering from lesions associated with multiple sclerosis, detecting subtle neurological impairments perceived by the patient [16]. The lower the score the fewer deficits are being perceived by the patient with a minimum of 0 and a maximum score of 80 points. The standardized questionnaire is provided in the supplement.

### Data Analysis

To test for group differences between subjects with and without lesions, a two-sample Student’s t test was used. Statistical significance threshold was set at *p* < 0.05. To test for correlation of DWI lesion count and examiner experience, we used a Spearman correlation analysis.

## Results

### Sample Characteristics

Demographical subject characteristics along with clinically reported cardiovascular risk factors and DSA-related data are presented in Table 1. A total of 82 subjects received MRI imaging after elective DSA. The mean age was 58.3 ± 14.7 years with 56 female and 26 male subjects. The most important comorbidities included a history of smoking in 28 subjects (34.1%) and arterial hypertension in 41 patients (50%).

**Table 1:** Demographical, clinical, and interventional data

Study size (*n* = 82)	Mean	SD	Range	*n*
Sex (male/female)				26/56
Age (years)	58.3	± 14.7	17.3–90.3	
History of smoking				28
Arterial hypertension				41
Arteriosclerosis/Plaques				33
DWI lesions	0.7	± 1.9	0–14	54
Lesion volume in mm^3^	120.6	± 159.5	11.8–596.2	
Fluoroscopy duration (min)*	4.98		0.5–41.5	
No. of series	7.5	± 3.3	2–15	
No. of vessels probed	2.7	± 1.1	1–5	
Contrast used (ml)	55.4	± 24.0	14–115	
Examiner experience	2.1	± 0.9	1–3	

*median of *n* = 45

*DWI* diffusion-weighted imaging; *SD* standard deviation

### Incidence of DWI Lesions After DSA and Longitudinal Signal Characteristics

**Fig. 1:**
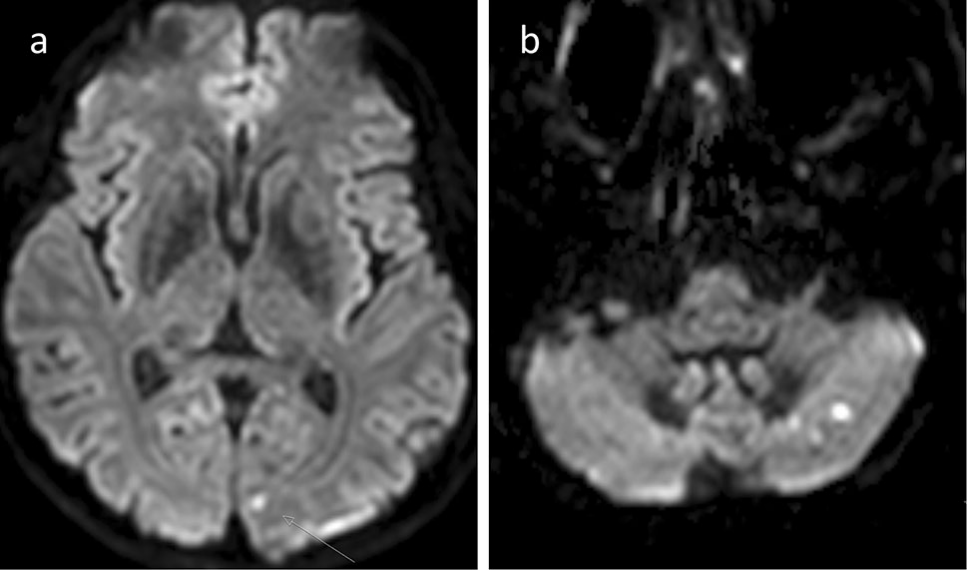
Typical appearance of microembolic brain lesions. **a** Diffusion-weighted imaging (DWI) of subject No. 2. A typical bright round-shaped diffusion-restricted lesion in the left cuneus. **b** DWI of subject No. 77. Three lesions of typical round-shape and different sizes in close proximity in the left cerebellar lobe

**Fig. 2:**
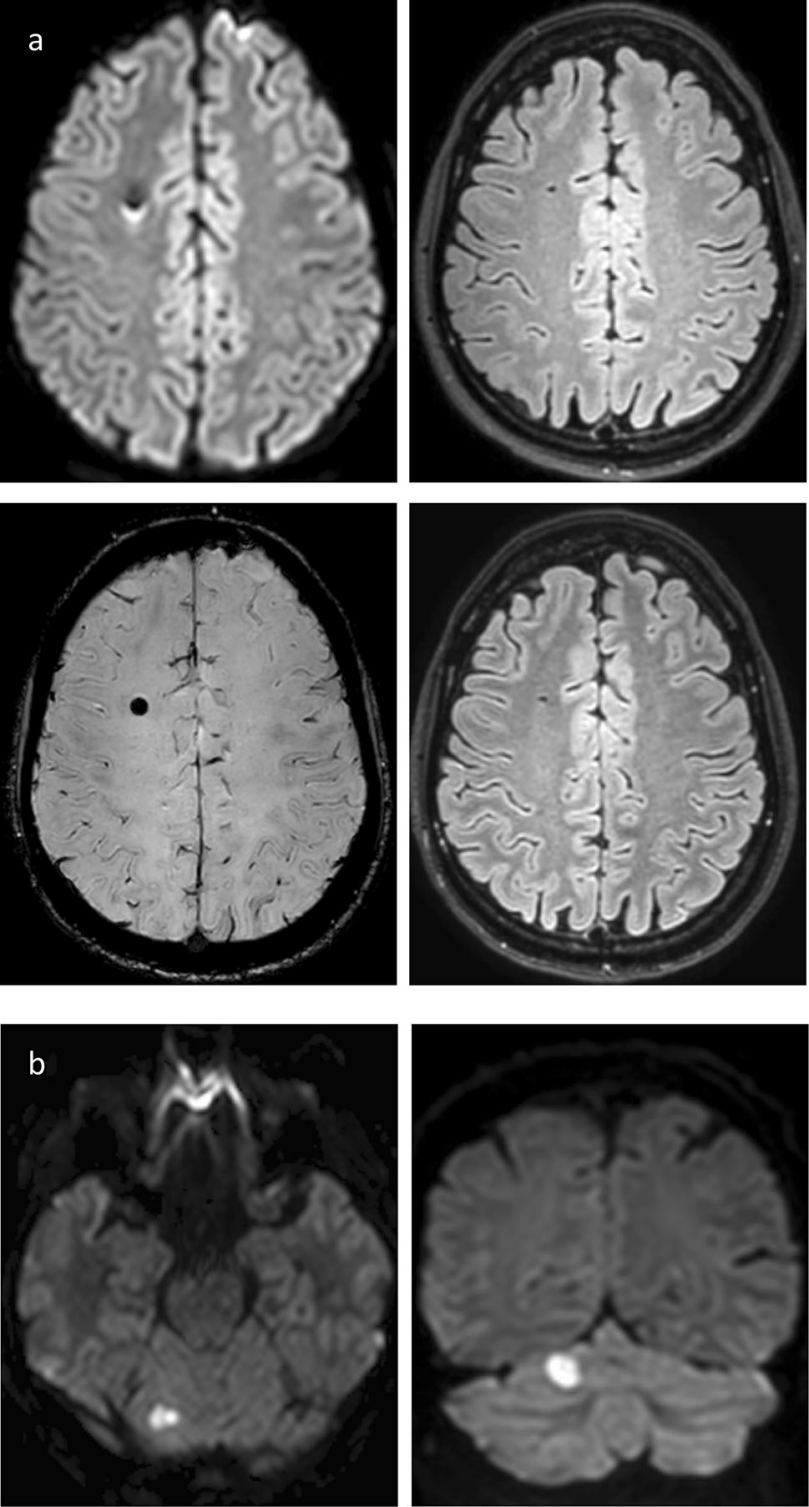
Atypical appearance of intracranial lesions following diagnostic DSA. **a** Subject No. 4. Upper row shows DWI (left) and FLAIR (right) immediately after DSA. Bottom row shows SWI (left) and FLAIR (right) at follow-up appointment. A lesion of central low DWI signal and a bright posterior rim with corresponding low signal in FLAIR can be seen after DSA. Follow-up shows high susceptibility with blooming in SWI and predominantly unchanged characteristics in FLAIR 7.7 months after DSA. **b** Subject No. 76. Axial DWI (left) and coronary DWI (right) after DSA show a roundish and unusually large DWI bright diffusion-restricted lesion in the right upper cerebellar lobe. Subject was lost to follow-up. FLAIR, Fluid attenuated inversion recovery. DSA, Digital subtraction analysis. DWI, Diffusion-weighted imaging. No, Number. SWI, Susceptibility-weighted imaging

Among the 82 subjects studied, 23 subjects (28.0%) showed a total number of 54 diffusion-restricted lesions following DSA when using high-resolution diffusion-weighted MRI (Table 1). Mean lesion size was 120.6 ± 159.5mm^3^. All lesions occurred with respect to the probed vessels´ vascular territories. The typical embolic like dot-shaped lesion appearance is demonstrated in Fig. 1. Figure 2 shows a case with a less common but larger ischemic lesion in the cerebellum. Despite the larger ischemic lesion volume, the subject showed no subjective symptoms or measurable deficits. Figure 2 also depicts an example of a subject with a lesion showing high susceptibility on SWI imaging which must have occurred during the procedure, as this subject had a previous MR scan immediately prior to the DSA showing no lesion.

**Fig. 3:**
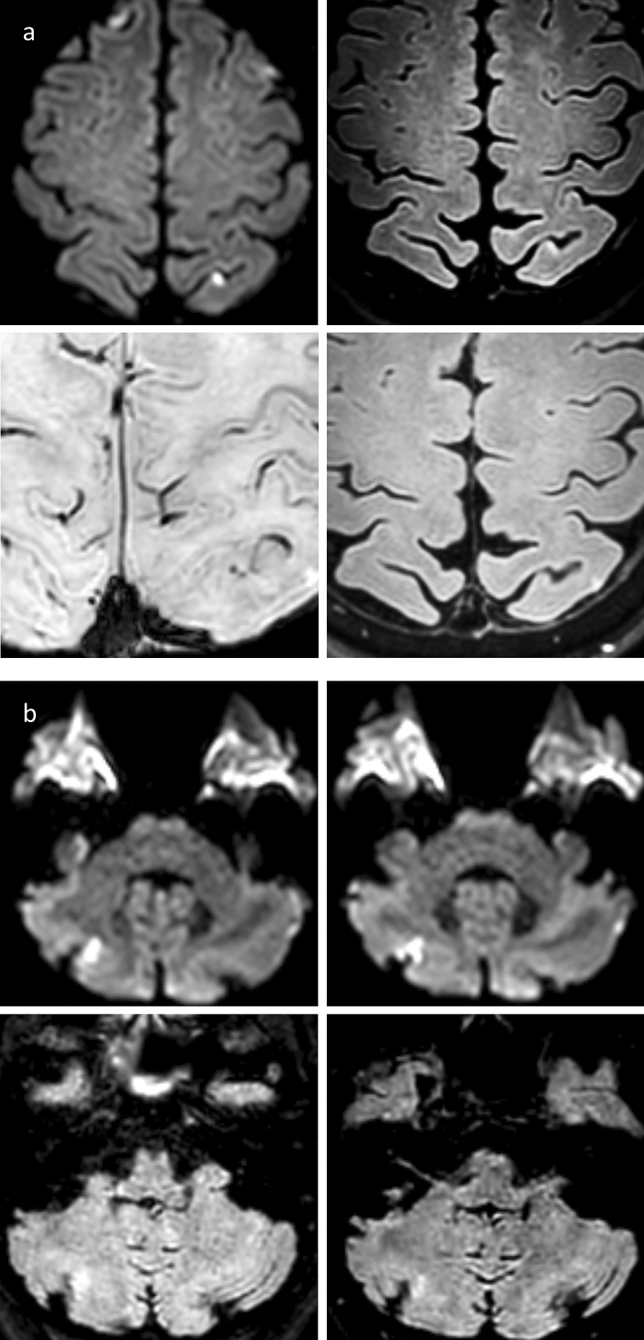
Persistent high-signal lesions at follow-up. **a** Subject No. 24. Upper row shows DWI (left) and slightly zoomed-in FLAIR (right) after DSA. Bottom row shows zoomed-in FLAIR (right) and even more zoomed-in SWI (left) at follow-up. DWI after DSA shows high signal corresponding to diffusion restriction in left superior parietal lobule. Diffusion-restricted area shows slight FLAIR hyperintensity following DSA and at follow-up with punctate low-signal in SWI. **b** Subject No. 45. Upper row shows DWI at different heights after DSA with a high-signal diffusion-restricted cortical lesion of the right cerebellar lobe. Bottom row shows FLAIR after DSA (left) and at follow-up (right) with well visible persistent FLAIR hyperintensity at both time appointments. Abbreviations: FLAIR, fluid attenuated inversion recovery; DSA, digital subtraction analysis; DWI, diffusion-weighted imaging; No, number; SWI, susceptibility-weighted imaging

A total of 12 subjects out of all 23 subjects presenting with lesions at baseline, received follow-up MRI examinations to track total of 25 lesions (Table 2). Due to non-participation and one case of unrelated death, 11 subjects were lost to follow-up. Follow-up scans were performed a median of 5.1 months after the intervention. Five of these 25 lesions (20%) remained visible on FLAIR imaging as hyperintense lesions at follow-up (Table 2). Figure 3 illustrates cases with persisting high FLAIR-signal at follow-up. Two subjects each showed one lesion with visibly increased susceptibility on SWI (8%).

**Table 2:** Follow-up data

Study size (*n* = 12)	Mean	SD	Range
FLAIR lesions (*n* = 5)	0.3	± 0.65	0–2
SWI lesions (*n* = 2)	0.4	± 0.5	0–1
Weeks after angiography	31.7	± 34.1	4–133
Fluoroscopy duration (min)	8.3^a^		0.5–41.5
No. of series	7.6	± 3.2	2–15
No. of vessels probed	2.7	± 1.1	1–6
Contrast used (ml)	55.9	± 24.0	14–115

*FLAIR* fluid attenuated inversion recovery, *SD* standard deviation, *SWI* susceptibility-weighted imaging

^*a*^median

### Group Comparison of Subjects with and Without Lesions

Group comparisons of subjects with visible lesions on postinterventional MRI compared to subjects without lesions are shown in Table 3. Subjects with lesions were significantly older (64.3 vs. 55.9, *p* = 0.021) compared to subjects without lesions, had more vessels probed (3.0 vs. 2.5, *p* = 0.035), received more fluoroscopy series (8.8 vs. 7.0, *p* = 0.026) and showed nonsignificantly longer fluoroscopy time (8.85 vs. 4.46 min, *p* = 0.252). Although more contrast medium was used in subjects with lesions, this difference was not statistically significant (62.9 vs. 52.5 ml, *p* = 0.079). A clinical history of arterial hypertension and visible plaques both resulted in significantly more DWI lesions on postinterventional MRI (0.8 vs. 0.4, *p* < 0.001 and 0.7 vs. 0.3, *p* = 0.004, respectively). A history of smoking was not significantly associated with more DWI lesions on postinterventional MRI (0.4 vs. 0.3, *p* = 0.558).

**Table 3:** Group comparison of subjects with and without lesions

Study size (*n* = 82)	No lesion (*n* = 59)	Lesion(s) (*n* = 23)	Significance (*p*)
Age (years)	55.9 ± 14.7	64.3 ± 13.0	**0.021**
Fluoroscopy duration (min)	4.46^a^	8.85^b^	0.252
No. of series	7.0 ± 3.2	8.8 ± 3.0	**0.026**
No. of vessels probed	2.5 ± 1.1	3.0 ± 0.8	**0.035**
Contrast used (ml)	52.5 ± 24.5	62.9 ± 21.5	0.079
Arterial hypertension	0.4 ± 0.5	0.8 ± 0.4	** < 0.001**
Arteriosclerosis/plaques	0.3 ± 0.5	0.7 ± 0.5	**0.004**
History of smoking	0.3 ± 0.5	0.4 ± 0.5	0.558

Group-level analysis for subjects with visible diffusion-restricted lesions on postinterventional MRI compared to subjects without lesions. Bold letters indicate statistical significance defined as *p* < 0.05. Standard deviation is given for each value indicated by ±

*No* number

^a^median of *n* = 38

^b^median of* n* = 7

**Fig. 4:**
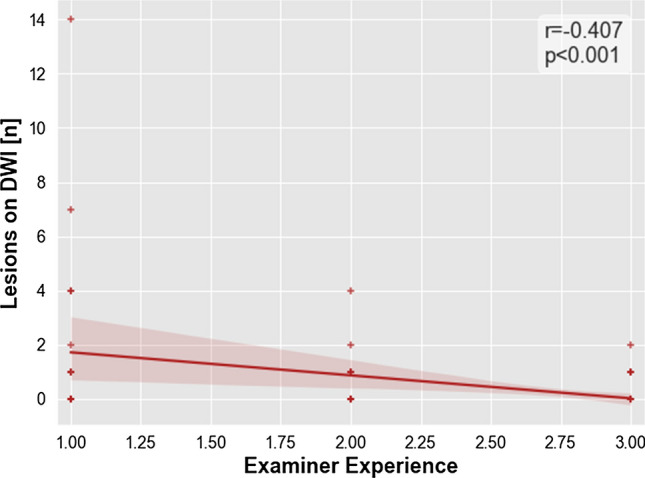
Relationship between the examiner’s degree of experience and microembolic lesions. Association between the examiner’s degree of experience—divided into three levels for assistant physician, specialist, and senior physician—and amount of microembolic lesions on DWI after DSA is shown as scatter plot. Examiner’s degree of experience is plotted on the x-axes and DWI lesions are plotted on the y-axes. Linear regression line, *p* value, and Spearman rho (*r*_*s*_) were added. Abbreviations: DSA, digital subtraction analysis; DWI, diffusion-weighted imaging

Using a Spearman correlation analysis between examiner experience and lesion presence revealed a significant negative correlation (*r* = − 0.407, *p* < 0.001, Fig. 4).

### Neurological Complications and Perceived Deficits

No deficits were shown before or after DSA as documented by a clinical neurological assessment. There was no significant difference in the investigator-guided standardized PDQ questionnaire mean score before angiography (13.3 ± 11.2) and after angiography (13.4 ± 11.8; *p* = 0.8). There was a nonsignificant increase of perceived deficits at the follow-up (17.8 ± 4.4.) compared to baseline (*p* = 0.09, Table 4).

**Table 4:** Perceived deficits questionnaire (PDQ)

Study size (*n* = 46)	Mean	SD	Range
Before intervention	13.3	± 11.2	0–47
Post intervention	13.4	± 11.8	0–47
At follow-up*	17.8	± 4.4	11–23

Subjects received an investigator-guided normative questionnaire prior to diagnostic digital subtraction analysis (DSA), after DSA, and at the follow-up appointment. Deficits are evaluated with a minimum score of 0 and a maximum score of 80 points with lower values indicating fewer deficits

*SD* standard deviation

*n = 6

## Discussion

This study showed that silent ischemic lesions after cerebral DSA occur in 28% of subjects as depicted by high-resolution isotropic DWI. This is consistent with previous studies that reported DWI lesions in up to 40% of patients after DSA [5–11, 17]. Nevertheless, the current rate of 28% is relatively high considering that heparinized saline drips were used and that only diagnostic DSA procedures short in duration were part of the study. This relatively high incidence of lesions could be partly explained by the methodological differences between our study and previous ones. We used a high-quality modern 3 Tesla MRI scanner with DWI sequences 2 mm isotropic resolution acquired in two axes (axial and coronal slices). This is a great improvement over previous studies that only had 1.5 Tesla MRI scanners with low-resolution DWI sequences with slice thicknesses of 5–6 mm, if MRI-related data were presented in the manuscript at all. Furthermore, our study was conducted prospectively, thus excluding confounding factors typically associated with retrospective studies. Despite the small size of the DWI lesions, they were still visible in 25% of subjects after a mean of 8 months.

Significant risk factors for lesion occurrence in our study were both examiner-independent and -dependent. Subject age and vascular risk factors, such as arterial hypertension or visible plaques on CT, were associated with DWI lesions, supporting the assumption of an association of vascular risk factors with silent DWI lesions [13]. Interestingly, in our study, the amount of contrast agent used was not significantly correlated with lesion occurrence, although previous Doppler sonographic studies showed a particularly high number of embolic signals during contrast injection [12, 18]. This indicates that complications during contrast injection, such as air bubbles or microparticles contained in the agent, presumably play an inferior role compared to vascular disease or intravascular microclot formation. Mechanical vessel wall stress may play a more important role in lesion development. This is further strengthened by recent findings that DWI lesions are more frequent when a transradial versus a transfemoral approach is used [19].

Furthermore, examiner experience inversely correlated with the number of DWI lesions, i.e., DSA performed by less experienced examiners resulted in more DWI lesions, and the number of probed vessels was significantly higher in patients with DWI lesions. This indicates that increased friction and longer duration of catheterization may cause microclots or loosen vessel adherent embolic material that forms as part of arteriosclerosis.

### Clinical Implication of DWI Lesion Occurrence

Relevant neurological complications after DSA are rare, [1–4, 14, 20, 21] and indeed no measurable neurological complications were observed in our study. It is not conclusively understood why the number of DWI lesions greatly exceeds the apparent neurological complications. It may be a combination of localization in a non-eloquent area, small infarct volume, and limited neurological-psychiatric systematic follow-up [5, 13]. Nevertheless, it is known that covert strokes increase the long-term risk of cognitive decline, subtle visual field defects, or depressive symptoms, which are not routinely assessed in detail after DSA in clinical practice [4, 6, 11, 13, 14]. In this study, we used a structured questionnaire to detect subtle changes subjectively perceived by the patient before, immediately after, and several months after DSA. There was no change in the mean score after DSA, and a nonsignificant increase at the follow-up visit.

Although a significant benefit in DWI lesion occurrence from patient-specific simulation training the day before DSA has not been demonstrated in a one-time experiment, efforts must be made to optimize training including angiography simulators within a structured curriculum to reduce the modifiable risk of the examiner’s skill level [22]. In addition, there is evidence of a reduction in DWI lesion load by embolic protection devices such as air filters, but their functional benefit has not been demonstrated to date [13, 23]. Randomized trials or even expert consensus have been lacking, resulting in nonuniformly performed DSA [13]. High-resolution DWI with a structured prospective longitudinal assessment, as in our study, could serve as a tool to compare and evaluate different angiography techniques or devices.

## Limitations

The results of this study are relevant because it is based on a healthy cohort and diagnostic DSA. More complex DSA interventions such as neurointerventional procedures were not part of this study and may potentially be associated with a higher lesion occurrence. Furthermore, the results should be interpreted with caution as the sample size of the follow-up cohort is comparatively small, the PDQ is primarily validated for patients with multiple sclerosis, and the self-perceived increase in deficits was not significant.

## Conclusion

Diagnostic cerebral DSA is associated with a considerable number of partially persistent DWI lesions when prospectively examined by high-resolution modern MRI. Although no measurable, significant clinical, or self-perceived complications have been observed in this cohort, further efforts should be made to better understand the impact of these lesions and improve the examiner’s skill level, especially in patients with vascular disease.

## Supplementary Information

Below is the link to the electronic supplementary material.

Supplementary file1 (PDF 134 KB)

## References

[1] Olivecrona H (1977). Complications of cerebral angiography. Neuroradiology.

[2] Earnest Ft, Forbes G, Sandok BA (1984). Complications of cerebral angiography: prospective assessment of risk. AJR Am J Roentgenol.

[3] Heiserman JE, Dean BL, Hodak JA (1994). Neurologic complications of cerebral angiography. AJNR Am J Neuroradiol.

[4] Mani RL, Eisenberg RL (1978). Complications of catheter cerebral arteriography: analysis of 5000 procedures. III. Assessment of arteries injected, contrast medium used, duration of procedure, and age of patient. AJR Am J Roentgenol.

[5] Bendszus M, Koltzenburg M, Burger R, Warmuth-Metz M, Hofmann E, Solymosi L (1999). Silent embolism in diagnostic cerebral angiography and neurointerventional procedures: a prospective study. The Lancet.

[6] Britt PM, Heiserman JE, Snider RM, Shill HA, Bird CR, Wallace RC (2000). Incidence of postangiographic abnormalities revealed by diffusion-weighted MR imaging. AJNR Am J Neuroradiol.

[7] Chuah KC, Stuckey SL, Berman IG (2004). Silent embolism in diagnostic cerebral angiography: detection with diffusion-weighted imaging. Australas Radiol.

[8] Hahnel S, Bender J, Jansen O (2001). Clinically silent cerebral embolisms after cerebral catheter angiography. Rofo.

[9] Laible M, Mohlenbruch M, Horstmann S (2017). Peri-procedural silent cerebral infarcts after left atrial appendage occlusion. Eur J Neurol.

[10] Markus H, Loh A, Israel D, Buckenham T, Clifton A, Brown MM (1993). Microscopic air embolism during cerebral angiography and strategies for its avoidance. Lancet.

[11] Sato M, Nakai Y, Tsurushima H, Shiigai M, Masumoto T, Matsumura A (2013). Risk factors of ischemic lesions related to cerebral angiography and neuro-interventional procedures. Neurol Med Chir.

[12] Dagirmanjian A, Davis DA, Rothfus WE, Deeb ZL, Goldberg AL (1993). Silent cerebral microemboli occurring during carotid angiography: frequency as determined with doppler sonography. AJR Am J Roentgenol.

[13] Goyal M, Ganesh A, Tymianski M, Hill MD, Ospel JM (2021). Iatrogenic diffusion-weighted imaging lesions. Stroke.

[14] Dion JE, Gates PC, Fox AJ, Barnett HJ, Blom RJ (1987). Clinical events following neuroangiography: a prospective study. Stroke.

[15] Kruis RW, Vlasveld FA, Van Dijk D (2010). The (un)importance of cerebral microemboli. Semin Cardiothorac Vasc Anesth..

[16] Sullivan MJ, Edgley K, Dehoux E. A survey of multiple sclerosis: I. Perceived cognitive problems and compensatory strategy use. Can J Rehabil. 1990;

[17] Gerraty RP, Bowser DN, Infeld B, Mitchell PJ, Davis SM (1996). Microemboli during carotid angiography. Stroke.

[18] Park KY, Chung PW, Kim YB, Moon HS, Suh BC, Yoon WT (2011). Post-interventional microembolism: cortical border zone is a preferential site for ischemia. Cerebrovasc Dis.

[19] Carraro do Nascimento V, de Villiers L, Hughes I, Ford A, Rapier C, Rice H. Transradial versus transfemoral arterial approach for cerebral angiography and the frequency of embolic events on diffusion weighted MRI. J Neurointerv Surg. 2022; 10.1136/jnis-2022-019009 35868855

[20] Wojak JC, Abruzzo TA, Bello JA (2015). Quality Improvement Guidelines for Adult Diagnostic Cervicocerebral Angiography: Update Cooperative Study between the Society of Interventional Radiology (SIR), American Society of Neuroradiology (ASNR), and Society of NeuroInterventional Surgery (SNIS). J Vasc Interv Radiol.

[21] Dawkins A, Evans AL, Wattam J (2007). Complications of cerebral angiography: a prospective analysis of 2,924 consecutive procedures. Neuroradiology.

[22] Kreiser K, Gehling K, Ströber L, Zimmer C, Kirschke J (2020). Simulation training in neuroangiography: transfer to reality. CardioVasc Interv Radiol.

[23] Bendszus M, Stoll G (2006). Silent cerebral ischaemia: hidden fingerprints of invasive medical procedures. Lancet Neurol.

