# Supplementary material for: Incidence, Clinical Significance, and Longitudinal Signal Characteristics of Ischemic Lesions Related to Diagnostic Cerebral Catheter Angiography
Source: Cardiovasc Intervent Radiol. 2023 Mar 29;46(7):921–8. doi: 10.1007/s00270-023-03415-z (PMC10322964; doi:10.1007/s00270-023-03415-z)
Supplement: Supplementary file 1 — Supplementary file1 (PDF 134 KB) [file 270_2023_3415_MOESM1_ESM.pdf]

Supplementary Material for:

Incidence, clinical significance, and longitudinal signal characteristics of ischemic lesions related to diagnostic cerebral catheter angiography

David Schinz[1], Thomas Zimmermann[1], Jens Göttler[1], Dominik Sepp[1], Claus Zimmer[1], Tobias Boeckh-Behrens[1], Jan S. Kirschke[1], Kornelia Kreiser[1], Hans Liebl[1]

[1] Department of Diagnostic and Interventional Neuroradiology, Klinikum rechts der Isar, Technische Universität München, Munich, Germany

[2]Department of Radiology/Neuroradiology, RKU, Universitäts- und Rehabilitationskliniken Ulm, gGmbH, Oberer Eselsberg 45, 89081, Ulm, Germany

Corresponding Author:

David Schinz david.schinz@tum.de

Department of Diagnostic and Interventional Neuroradiology, Klinikum rechts der Isar, Technische Universität München, Ismaninger Str. 22, 81675 Munich, Germany

Phone: +49 89 4140 4651, Fax: +49 89 4140 4887

Supplement Methods

Self-perceived deficits testing

The Perceived Deficits Questionnaire (PDQ) was conducted by an examiner (specifically trained medical student) in German language. The questionnaire was developed in English language for the assessment of perceived cognitive deficits from the patient's perspective by Sullivan, Edgley, and Dehoux.[1] The PDQ was designed for the effects of multiple sclerosis, but tests a wide range of cognitive impairments and is part of the Multiple Sclerosis Quality of Life Inventory.

Every questionnaire was performed before digital subtraction angiography (DSA) and after DSA before dismissal from the institution. Patients with diffusion weighted imaging (DWI) lesions had to perform the test again on the day of the follow up examination.

The questions should be answered with regard to the last 4 weeks. Patients had to chose from four possible answers:

- Never (= 0 points)
- Rarely (= 1 point)
- Sometimes (= 2 points)
- Often (= 3 points)
- Almost every time (=4 points)

The points for all 20 questions were summed up and resulted in a total score for each patient. Thus minimum total score was 0, maximum total score was 80.

The PDQ:

How often did you… **Wie oft…**

1. lose your train of thought when speaking?	1. haben Sie Ihren Gedankengang beim Sprechen verloren?
2. have difficulty remembering the names of people, even the ones you have met several times?	2. hatten Sie Schwierigkeiten, sich die Namen von Personen zu merken, sogar solche, die Sie bereits häufiger getroffen haben?
3. forgot what you came into the room for?	3. haben Sie vergessen, weswegen Sie in ein Zimmer kamen?
4. have trouble getting things organized?	4. hatten Sie Schwierigkeiten, Dinge zu organisieren?
5. have trouble concentrating on what people are saying during a conversation?	5. hatten Sie Schwierigkeiten, sich auf das zu konzentrieren, was die Leute während eines Gesprächs sagen?
6. forget if you have already done something?	6. haben Sie vergessen, ob Sie etwas bereits erledigt haben?
7. miss appointments and meetings you had scheduled?	7. haben Sie Termine und Besprechungen versäumt, die Sie geplant hatten?

8. have difficulties planning what to do in the day?	8. hatten Sie Schwierigkeiten zu planen, was Sie am Tag tun sollten?
9. have trouble concentrating on things like watching a television program or reading a book?	9. hatten Sie Schwierigkeiten, sich auf Dinge wie z.B. die Fernsehsendung oder ein Buch zu konzentrieren?
10. forget what you did the night before?	10. haben Sie vergessen, was Sie am Abend zuvor getan haben?
11. forget the date unless you looked it up?	11. haben Sie das Datum vergessen, wenn Sie es nicht nachgeschlagen haben?
12. have trouble getting started, even if you had a lot of things to do?	12. hatten Sie Schwierigkeiten, Aufgaben zu beginnen, obwohl Sie viel zu erledigen hatten?
13. find your minding drifting?	13. kam es vor, dass Ihre Gedanken schweiften?
14. forgot what you talked about after a telephone conversation?	14. haben Sie nach einem Telefonat vergessen, worüber Sie gesprochen haben?
15. forgot to do things like turn off the stove or turn on your alarm clock?	15. haben Sie vergessen den Herd auszuschalten oder den Wecker einzuschalten?

16. feel like your mind went totally blank?	16. hatten Sie das Gefühl, dass Ihre Gedanken völlig leer waren?
17. have trouble holding phone numbers in your head, even for a few seconds?	17. hatten Sie Schwierigkeiten, Telefonnummern im Kopf zu behalten, selbst für ein paar Sekunden?
18. forget what you did last weekend?	18. haben Sie vergessen, was Sie am vergangenen Wochenende getan haben?
19. forget to take your medication?	19. haben Sie vergessen, Ihre Medikamente einzunehmen?
20. have trouble making decisions?	20. hatten Sie Schwierigkeiten, Entscheidungen zu treffen?

1. Sullivan MJ, Edgley K, Dehoux E. A survey of multiple sclerosis: I. Perceived cognitive problems and compensatory strategy use. Canadian Journal of Rehabilitation 1990.
